# The Development of a Plant Risk Evaluation (PRE) Tool for Assessing the Invasive Potential of Ornamental Plants

**DOI:** 10.1371/journal.pone.0121053

**Published:** 2015-03-24

**Authors:** Christiana Conser, Lizbeth Seebacher, David W. Fujino, Sarah Reichard, Joseph M. DiTomaso

**Affiliations:** 1 Department of Plant Sciences, University of California Davis, Davis, California, United States of America; 2 Washington State Department of Ecology, Lacey, Washington, United States of America; 3 University of California Center for Urban Horticulture, Davis, California, United States of America; 4 University of Washington Botanical Gardens, Seattle, Washington, United States of America; Shandong University, CHINA

## Abstract

Weed Risk Assessment (WRA) methods for evaluating invasiveness in plants have evolved rapidly in the last two decades. Many WRA tools exist, but none were specifically designed to screen ornamental plants prior to being released into the environment. To be accepted as a tool to evaluate ornamental plants for the nursery industry, it is critical that a WRA tool accurately predicts non-invasiveness without falsely categorizing them as invasive. We developed a new Plant Risk Evaluation (PRE) tool for ornamental plants. The 19 questions in the final PRE tool were narrowed down from 56 original questions from existing WRA tools. We evaluated the 56 WRA questions by screening 21 known invasive and 14 known non-invasive ornamental plants. After statistically comparing the predictability of each question and the frequency the question could be answered for both invasive and non-invasive species, we eliminated questions that provided no predictive power, were irrelevant in our current model, or could not be answered reliably at a high enough percentage. We also combined many similar questions. The final 19 remaining PRE questions were further tested for accuracy using 56 additional known invasive plants and 36 known non-invasive ornamental species. The resulting evaluation demonstrated that when “needs further evaluation” classifications were not included, the accuracy of the model was 100% for both predicting invasiveness and non-invasiveness. When “needs further evaluation” classifications were included as either false positive or false negative, the model was still 93% accurate in predicting invasiveness and 97% accurate in predicting non-invasiveness, with an overall accuracy of 95%. We conclude that the PRE tool should not only provide growers with a method to accurately screen their current stock and potential new introductions, but also increase the probability of the tool being accepted for use by the industry as the basis for a nursery certification program.

## Introduction

The nursery and landscape industry has introduced over 50,000 ornamental species to the US [1–2]. The number of cultivars in the North America has increased rapidly from 29,000 in 1987 to 105,000 in 2008 [3]. Despite the large number of plant introductions, only a small percentage of them (between 0.1% and 1%) have become invasive [1–2, 4]. Invasive plants can be defined as introduced plants that escape cultivation and become dominant or cause harm in natural areas [2]. Sixty percent of the 214 invasive plants on the California Invasive Plant Council’s Invasive Plant Inventory were intentionally introduced for human uses, and 47% of those plants are landscape ornamentals [5–6]. In Florida the trend is similar, with 47% of the invasive plants originally introduced as ornamentals [7]. Throughout North America, 82% of the 235 invasive woody plants are horticultural in origin [8]. For the entire country, including both herbaceous and woody species, estimates of the invasive flora derived from the nursery industry vary widely from 34 to 83% [9].

The most cost effective way to prevent introduction of new invasive ornamental plants is prevention at the beginning of the nursery supply chain and before it has escaped into the environment [1, 10–12]. Risk assessment is the most accurate method of screening for invasive species in all pest categories (plants, animals, insects, and pathogens) [1, 13–15]. Weed Risk Assessment (WRA) is a systematic process that uses available evidence to estimate the risk of a plant species becoming invasive in a given region [15]. While many WRA tools exist for a variety of applications, including evaluating plants in botanical gardens [16], none were specifically designed to screen ornamental plants prior to being released into the environment.

The first WRA tool was developed in Australia for import screening purposes and has been adapted for use in other parts of the world [13, 17–25]. The 49-question Australian WRA tool has been shown to be 90 to 100% accurate in correctly identifying invasive plants, but results varied dramatically in its accuracy levels in categorizing known non-invasive plants, ranging from 21 to 75% accuracy. Thus, the tool is considered by many to be too conservative in predicting invasiveness, with many non-invasive species categorized as invasive. This will likely compromise its practical application in the horticultural industry [25–26]. The other existing WRA tools have limitations for the application of screening ornamentals plants for invasive risk in a practical way. Europe and the U.S. also have WRA tools to prevent the importation of invasive plants, but these tools screen for large geographic regions, which limits their application for screening ornamentals [15, 27–30]. The tool developed by the United States (US) Department of Agriculture Animal and Plant Health and Inspection Service, Plant Protection and Quarantine (USDA-APHIS-PPQ) has high accuracy in classifying major-invaders (94% accuracy), and higher accuracy for non-invaders (97% accuracy) [15], but is not designed for evaluating potential invasiveness on a regional scale or in determining invasive risk with plants in the early pre-marketing stages. These existing WRA tools all contain questions regarding the environmental impact of the plant species being evaluated. The majority of the time these questions cannot be answered for ornamental plants because they have not yet become weedy or invasive. Thus, there are no known environmental impacts for most species evaluated.

To be useful to the nursery and landscape industry, a WRA tool must contain questions that are answerable for ornamental plants and retain high accuracy in predicting both potential invasiveness and non-invasiveness. Because only a small number of introduced (non-native) ornamental plants become invasive (low base-rate of <1%) [4, 21, 31], we expected most non-native species would be categorized as having low invasive potential and a small proportion would have a high risk of becoming invasive [1]. Because of this low base-rate, risk assessment models have a higher probability of predicting some false positives (non-invasive species rejected as high risk for invasiveness) than false negatives (invasive species accepted as low risk for invasiveness) [21]. For WRA tools used for border screening, like the Australian model, this is acceptable because the economic impacts of accepting an invasive species (false negative) are much greater than the potential reduction in economic benefits from prohibiting the import of a non-invader [31]. As a result, most risk assessment tools tend to be more conservative in classifying invasiveness, and thus, less accurate in predicting non-invasiveness.

The objective of this study was to use a science-based and systematic process to develop a highly accurate (for both invasive and non-invasive plants) Plant Risk Evaluation tool (PRE) specifically for screening ornamental plants. We assessed questions from existing WRA tools and developed the PRE tool with the most predictive and relevant questions for ornamental plants. The tool was designed to possess the maximum overall accuracy (sensitivity and specificity) while making allowances for ease of use and brevity. The long-term goal of the project is to provide a voluntary screening tool for the horticultural industry that ultimately prevents new potentially invasive plants from being introduced or sold in regions of the US where they are likely to become invasive.

## Materials and Methods

The PlantRight campaign [32], coordinated by San Francisco environmental NGO Sustainable Conservation, developed the science-based Plant Risk Evaluation (PRE) tool to assist the horticultural industry in assessing ornamental plants for invasiveness. The PlantRight campaign is a collaboration of stakeholders from the nursery industry, government agencies, academic institutions, and environmental groups.

The PlantRight PRE tool relies on available scientific evidence to estimate the risk of an ornamental plant species becoming invasive and causing environmental or economic harm in a defined geographic region. For this study, we evaluated plants for invasiveness within the different climatic regions of California, but the PRE tool can be used to evaluate plants for any specified region. To avoid overestimating accuracy for plants known to be invasive within California, we excluded all information about invasion and impacts that was specific to the geographic area of the assessment (i.e., California).

In this paper we present the initial step in the PRE tool’s development, accuracy and validation as a proof of concept. Additional steps currently under evaluation further validate the model by 1) comparing the PRE tool and Australian’s WRA for consistency among multiple evaluators; 2) expanding the PRE tool validation for other regions of the United States; 3) developing and validating a PRE Rapid Screening (5-question) pre-assessment tool for handling large data sets; and 4) increasing the geographic predictability of the PRE tool by incorporating climatic matching maps using CLIMEX.

### Development of the Plant Risk Evaluation (PRE) tool

The initial step in developing the PRE tool required an evaluation of several pre-existing WRA screening tools [2, 15–17, 27, 33–34] to determine the most appropriate and highly predictive questions contributing to model accuracy for ornamental plants. We evaluated the existing WRA tools and selected questions that were found in two or more of these tools. Using these criteria we identified 56 questions to evaluate that ranged in topic from invasive history, climate match, difficulty of control, environmental impacts, reproductive and dispersal strategies, and growth rate (S1 Table). Each question was given the same weighted score used in the WRA tool it was taken from, with most questions having a score of 1 for a yes answer and 0 for a no answer.

We screened a small population of plant species (35 plants—21 known invasive and 14 known non-invasive plants) to do our initial evaluation of the 56 questions we selected from existing WRA models. While this sample size is relatively small, it served as a preliminary evaluation to test our criteria for including questions in the PRE tool and to determine if the questions were answerable for ornamental plants, which in many cases they were not. The invasive plants were selected from the California Invasive Plant Council’s (Cal-IPC) California Invasive Plant Inventory [6], a list of species that have known or suspected impacts in California and which represent diversity in both taxonomic relationship and phenology (Table 1). The Cal-IPC Inventory is based on an assessment that focuses on impacts and invasiveness of species already within the state, while WRA models typically consider species that are present but not yet invasive, or have yet to be introduced to a region. Thus, the behavior of the plant is more related to other regions of the country or world. The non-invasive species were chosen from the PlantRight’s *Suggested Alternatives for Invasive Garden Plants* [35] and the Cal-IPC *Don’t Plant a Pest Alternatives List* [36]. These lists of non-invasive plants are considered appropriate for California and are similar in form and function to known invasive plants.

**Table 1:** List of plants evaluated with 56 questions and their total score and percentage of questions answered.

**Family**	**Invasive species**	**Common name**	**Growth form**	**Total score**	**% questions answered**
Apiaceae	*Conium maculatum*	Poison-hemlock	Biennial dicot	30	89
Apiaceae	*Foeniculum vulgare*	Fennel	Perennial dicot	36	93
Asteraceae	*Carduus nutans*	Musk thistle	Biennial dicot	35	93
Asteraceae	*Cotula coronopifolia*	Brassbuttons	Perennial dicot	20	80
Asteraceae	*Delairea odorata*	Cape-ivy	Perennial dicot	31	88
Asteraceae	*Silybum marianum*	Blessed milkthistle	Annual dicot	33	88
Brassicaceae	*Sisymbrium irio*	London rocket	Annual dicot	21	89
Chenopodiaceae	*Salsola paulsenii*	Barbwire Russian-thistle	Annual dicot	30	91
Dipsacaceae	*Dipsacus sativus*	Fuller's teasel	Biennial dicot	32	91
Euphorbiaceae	*Triadica sebifera*	Chinese tallowtree	Tree	31	86
Fabaceae	*Acacia melanoxylon*	Black acacia	Tree	33	93
Fabaceae	*Genista monspessulana*	French broom	Shrub	37	91
Myrtaceae	*Eucalyptus camaldulensis*	Red gum	Tree	30	95
Myrtaceae	*Eucalyptus globulus*	Tasmanian blue gum	Tree	33	91
Poaceae	*Aegilops triuncialis*	Barb goatgrass	Annual grass	28	82
Poaceae	*Stipa capensis* (= *Achnatherum capense*)	Mediterranean steppegrass	Annual grass	21	84
Polygonaceae	*Polygonum cuspidatum*	Japanese knotweed	Shrub	27	84
Rosaceae	*Rubus armeniacus*	Himalaya blackberry	Shrub	44	98
Salviniaceae	*Salvinia molesta*	Giant salvinia	Perennial fern	30	93
Scrophulariaceae	*Linaria genistifolia* ssp. *dalmatica*	Dalmatian toadflax	Perennial dicot	32	89
Tamaricaceae	*Tamarix parviflora*	Smallflower tamarisk	Tree/shrub	33	95
			***Average***	***31***	***90***
**Family**	**Non-Invasive species**	**Common name**		**Total score**	**% questions answered**
Fabaceae	*Calliandra tweedii*	Brazilian flamebush	Shrub	9	86
Campanulaceae	*Campanula poscharskyana*	Serbian bellflower	Perennial dicot	11	91
Bignoniaceae	*Chilopsis linearis*	Desert willow	Tree	11	91
Cupressaceae	*Cupressus arizonica*	Arizona cypress	Tree	11	89
Aizoaceae	*Delosperma cooperi*	Trailing iceplant	Perennial dicot	14	88
Asteraceae	*Euryops pectinatus*	Yellow bush daisy	Shrub	13	86
Oleaceae	*Forsythia* x *intermedia*	Forsythia	Shrub	5	95
Poaceae	*Helictotrichon sempervirens*	Blue oatgrass	Perennial grass	6	89
Rosaceae	*Kerria japonica*	Japanese rose	Shrub	10	88
Lauraceae	*Laurus nobilis*	Sweet bay	Tree	7	88
Agavaceae	*Phormium tenax*	New Zealand flax	Perennial monocot	11	89
Buxaceae	*Sarcococca hookeriana* var. *humilis*	Sweet box	Shrub	7	88
Lamiaceae	*Teucrium chamaedrys*	Wall germander	Perennial dicot	10	89
Caprifoliaceae	*Viburnum awabuki*	Sweet viburnum	Tree	9	88
			***Average***	***10***	***89***

For each plant species evaluated, a complete literature review was performed, along with searches of online databases and species fact sheets. The information gathered in the literature and internet review was used to answer yes or no for each question in the model. If no information was available to answer a specific question, that question was answered with a “?”, meaning “unknown”.

For each plant species evaluated, we calculated the total score (the sum of points from all the questions) and the percentage of questions that were answered (Table 1). To determine which questions contributed to the predictability of invasiveness and non-invasiveness, we used a two-tailed Fischer’s Exact Test (S1 Table). For each question, we calculated the percentage of times it was answered for all known invasive and non-invasive plants. We determined which questions to eliminate based on the following criteria: 1) Fischer’s Exact Test (two-tailed) *P<0*.*05*, 2) question were answered <20% of the time, and 3) question was irrelevant or biased for ornamental plants. Through this process, we developed the PRE tool by narrowing the 56 questions down to 19 (Table 2).

**Table 2:** PRE tool questions and their statistical predictability in separating known invasive and non-invasive species.

Question #	Question in PRE tool	Fisher's Exact Test (2-tail)	% Q was answered for invasive plants	% Q was answered for non-invasive plants	Point values Yes/No
1	Has the species become naturalized where it is not native?^[^ ^15^ ^],[^ ^17^ ^],[^ ^27^ ^],[^ ^33^ ^]^	*P* <0.0001*	100	100	1/0
2	Is the species noted as being invasive elsewhere in the US or world?^[^ ^2^ ^],[^ ^15^ ^],[^ ^16^ ^],[^ ^33^ ^]^	*P* <0.0001*	100	100	2/0
3	Is the species noted as being invasive elsewhere in the US or world in a similar climate?^[^ ^2^ ^],[^ ^15^ ^],[^ ^16^ ^],[^ ^27^ ^],[^ ^33^ ^]^	*P* <0.0001*	100	100	3/0
4	Are other species of the same genus invasive in other areas with a similar climate?^[^ ^2^ ^],[^ ^15^ ^],[^ ^16^ ^],[^ ^33^ ^]^	*P* <0.0001*	100	100	1/0
5	Is the species found predominately in a climate that matches those within the region of introduction?^[^ ^15^ ^],[^ ^17^ ^],[^ ^27^ ^]^	—-	96	100	2/0
6	Does this plant displace native plants and dominate the plant community in areas where it has invaded? native.^[^ ^15^ ^],[^ ^16^ ^],[^ ^27^ ^],[^ ^33^ ^]^ Does this plant overtop and/or smother surrounding vegetation?^[^ ^15^ ^],[^ ^16^ ^],[^ ^17^ ^],[^ ^33^ ^]^	*P* <0.0001*	100	100	1/0
7	Is the plant noted as being highly flammable and/or promotes fire and/or changes fire regimes?^[^ ^15^ ^],[^ ^16^ ^],[^ ^17^ ^],[^ ^33^ ^]^	*P* <0.0001*	79	97	1/0
8	Is the plant a health risk to humans or animals/fish? (Toxic tendencies)^[^ ^15^ ^],[^ ^16^ ^],[^ ^17^ ^],[^ ^27^ ^],[^ ^33^ ^]^ Has the species been noted as impacting grazing systems?^[^ ^15^ ^],[^ ^17^ ^],[^ ^27^ ^]^	*P* = 0.0001*	100	100	1/0
9	Does the plant produce impenetrable thickets, blocking or slowing movement?^[^ ^15^ ^],[^ ^16^ ^],[^ ^17^ ^],[^ ^33^ ^]^	*P* = 0.0002*	93	100	1/0
10	Does this plant reproduce vegetatively via root sprouts/suckers^[^ ^2^ ^],[^ ^17^ ^]^ or stem/trunk sprouts/coppicing?^[^ ^2^ ^],[^ ^15^ ^]^	*P* = 0.0314*	98	100	1/0
11	Are fragments from this plant capable of producing new plants?^[^ ^2^ ^],[^ ^17^ ^]^	*P* = 0.0002*	100	100	1/0
12	Does this species produce viable seed?	*P* = 0.0001*	100	100	1/0
13	Does this plant produces copious viable seeds each year (>1000)?^[^ ^2^ ^],[^ ^17^ ^]^	*P* <0.0001*	86	78	1/0
14	Does this plant produce seeds that germinate quickly (<2 months)?^[^ ^2^ ^],[^ ^17^ ^]^	*P* = 0.1296	75	68	1/0
15	Does this plant have a short juvenile period? Does it produce seed within first three years (herbaceous) or first five years (woody)?^[^ ^2^ ^],[^ ^17^ ^]^	*P* = 0.0078*	89	54	1/0
16	Does this plant have a long flowering period with seeds produced for >3 months each year?^[^ ^2^ ^],[^ ^17^ ^]^	*P* = 0.2320	86	86	1/0
17	Are this plant's propagules dispersed by mammals/insects or birds or via domestic animals?^[^ ^15^ ^],[^ ^16^ ^],[^ ^17^ ^]^	*P* <0.0001*	100	97	1/0
18	Are this plant's propagules dispersed by wind or water?^[^ ^15^ ^],[^ ^16^ ^],[^ ^17^ ^]^	*P* <0.0001*	98	97	1/0
19	Are this plant's propagules dispersed via agriculture, contaminated seed, farm equipment, vehicles or boats, or clothing/shoes?^[^ ^15^ ^],[^ ^16^ ^],[^ ^17^ ^],[^ ^33^ ^]^	*P* <0.0001*	100	94	1/0
		**Average**	**97**	**97**	Range of 23/0

Fisher's Exact Test compared the 57 invasive species against the 37 non-invasive species for each question. Percent of each question (Q) answered is also included. Brackets after question indicate citation were question is included in WRA model.

### Accuracy testing of the Plant Risk Evaluation (PRE) tool

In a more robust evaluation, we tested the 19 question PRE tool by screening 92 additional plants (56 known invasive and 36 known non-invasive plants). Nearly all the known invasive plants were chosen from Cal-IPC California Invasive Plant Inventory [6] or are newly invasive in California (e.g., *Buddleja davidii* and *Billardiera heterophylla*) and represent a wide range of taxonomic families and growth forms (Table 3). The non-invasive species were selected from the UC Davis *Arboretum All-Star Plant List* [37] and PlantRight’s *Suggested Alternatives for Invasive Garden Plants* [35]. These plants also represent a wide range of growth forms and taxonomic groups (Table 3). The non-invasive species in both lists have many outstanding qualities for use in California gardens, including ease of propagation, few pest or disease problems, and high water use efficiency.

**Table 3:** Known invasive and non-invasive species evaluated with the PRE tool, including their scores and classification as either Reject, Needs Further Evaluation, or Accept.

Family	Species	Growth form	Classification	PRE score
***Known Invasive Species***				
Aizoaceae	*Carpobrotus chilensis*	Perennial dicot	Needs Further Evaluation	13
Aizoaceae	*Carpobrotus edulis*	Perennial dicot	Reject	17
Aizoaceae	*Mesembryanthemum crystallinium*	Perennial dicot	Reject	20
Anacardiaceae	*Schinus terebinthifolius*	Tree/shrub	Reject	18
Apocynaceae	*Vinca major*	Shrub	Reject	16
Araliaceae	*Hedera canariensis*	Shrub	Reject	16
Araliaceae	*Hedera helix*	Shrub	Reject	18
Asteraceae	*Acroptilon repens*	Perennial dicot	Reject	15
Asteraceae	*Ageratina adenophora*	Perennial dicot	Reject	15
Asteraceae	*Arctotheca calendula*	Perennial dicot	Reject	14
Asteraceae	*Centaurea calcitrapa*	Perennial dicot	Reject	18
Asteraceae	*Centaurea solstitialis*	Annual dicot	Reject	20
Asteraceae	*Cirsium vulgare*	Biennial dicot	Reject	19
Asteraceae	*Glebionis coronarium (= Chrysanthemum coronorium)*	Annual dicot	Reject	15
Asteraceae	*Leucanthemum vulgare*	Perennial dicot	Reject	20
Brassicaceae	*Brassica tournefortii*	Annual dicot	Reject	17
Brassicaceae	*Cardaria pubescens*	Perennial dicot	Reject	16
Brassicaceae	*Lepidium latifolium*	Perennial dicot	Reject	20
Buddlejaceae	*Buddleja davidii*	Shrub	Reject	18
Chenopodiaceae	*Halogeton glomeratus*	Annual dicot	Reject	15
Chenopodiaceae	*Salsola tragus*	Annual dicot	Reject	19
Clusiaceae	*Hypericum perforatum*	Perennial dicot	Reject	18
Elaeagnaceae	*Elaeagnus angustifolia*	Tree	Needs Further Evaluation	13
Fabaceae	*Acacia dealbata*	Tree/shrub	Reject	18
Fabaceae	*Cytisus scoparius*	Shrub	Reject	21
Fabaceae	*Cytisus striatus*	Shrub	Reject	18
Fabaceae	*Gleditsia triacanthos*	Tree	Needs Further Evaluation	13
Fabaceae	*Retama monosperma*	Shrub	Needs Further Evaluation	12
Fabaceae	*Spartium junceum*	Shrub	Reject	21
Geraniaceae	*Geranium robertianum*	Perennial dicot	Reject	18
Iridaceae	*Iris pseudacorus*	Perennial monocot	Reject	15
Lamiaceae	*Marrubium vulgare*	Perennial dicot	Reject	16
Lamiaceae	*Mentha pulegium*	Perennial dicot	Reject	18
Moraceae	*Ficus carica*	Tree	Reject	19
Myoporaceae	*Myoporum laetum*	Tree/shrub	Reject	17
Pittosporaceae	*Billardiera heterophylla* (= *Sollya heterophylla*)	Perennial vine/shrub	Reject	17
Poaceae	*Arundo donax*	Perennial grass	Reject	14
Poaceae	*Brachypodium sylvaticum*	Perennial grass	Reject	18
Poaceae	*Cortaderia jubata*	Perennial grass	Reject	20
Poaceae	*Cortaderia selloana*	Perennial grass	Reject	20
Poaceae	*Cynodon dactylon*	Perennial grass	Reject	20
Poaceae	*Ehrharta calycina*	Perennial grass	Reject	16
Poaceae	*Festuca arundinacea*	Perennial grass	Reject	18
Poaceae	*Pennisetum clandestinum*	Perennial grass	Reject	20
Poaceae	*Pennisetum setaceum*	Perennial grass	Reject	18
Poaceae	*Piptatherum miliaceum*	Perennial grass	Reject	17
Poaceae	*Saccharum ravennae*	Perennial grass	Reject	18
Pontederiaceae	*Eichhornia crassipes*	Perennial monocot	Reject	19
Ranunculaceae	*Ranunculus repens*	Perennial dicot	Reject	14
Rosaceae	*Cotoneaster franchetii*	Shrub	Reject	16
Rosaceae	*Cotoneaster pannosus*	Shrub	Reject	16
Rosaceae	*Crataegus monogyna*	Tree	Reject	16
Scrophulariaceae	*Linaria vulgaris*	Perennial dicot	Reject	21
Scrophulariaceae	*Verbascum thapsus*	Biennial dicot	Reject	16
Solanaceae	*Nicotiana glauca*	Tree	Reject	18
Tamaricaceae	*Tamarix ramosissima*	Tree/shrub	Reject	21
^**1**^ **TN**			**53**	** **
^**2**^ **FP**			**4**	
^**3**^ **IT**			**57**	
^**4**^ **Sensitivity for IP**			**93%**	
^**5**^ **Accuracy for IP**			**93%**	
***Known Non-Invasive Species***				
Aceraceae	*Acer rubrum*	Tree	Accept	10
Anacardiaceae	*Pistacia chinensis*	Tree	Accept	8
Apocynaceae	*Trachelospermum asiaticum*	Shrub	Accept	2
Asteraceae	*Tagetes lemmonii*	Perennial dicot/shrub	Accept	4
Caprifoliaceae	*Lonicera standishii*	Shrub	Accept	8
Caryophyllaceae	*Cerastium tomentosum*	Perennial dicot	Accept	10
Cistaceae	*Halimium lasianthum*	Shrub	Accept	2
Cornaceae	*Nyssa sylvatica*	Tree	Accept	4
Crassulaceae	*Sedum palmeri*	Perennial dicot	Accept	5
Cupressaceae	*Taxodium distichum*	Tree	Accept	4
Fabaceae	*Acacia boormanii*	Shrub	Accept	9
Fabaceae	*Cassia leptophylla*	Tree	Accept	9
Ginkgoaceae	*Ginkgo biloba*	Tree	Accept	4
Goodeniaceae	*Scaevola aemula*	Shrub	Accept	5
Griseliniaceae	*Griselinia littoralis*	Tree	Accept	4
Hamamelidaceae	*Liquidambar styraciflua*	Tree	Accept	7
Lamiaceae	*Salvia greggii*	Perennial dicot/shrub	Accept	3
Lamiaceae	*Salvia microphylla*	Shrub	Accept	4
Liliaceae	*Dasylirion wheeleri*	Shrub	Accept	7
Liliaceae	*Zephyranthes candida*	Perennial monocot	Accept	6
Myoporaceae	*Myoporum parvifolium*	Shrub	Accept	6
Myrtaceae	*Agonis flexuosa*	Tree	Accept	7
Oleaceae	*Chionanthus retusus*	Tree	Accept	5
Oleaceae	*Jasminum nudiflorum*	Perennial dicot	Accept	4
Plumbaginaceae	*Ceratostigma plumbaginoides*	Perennial dicot	Accept	6
Poaceae	*Bambusa multiplex*	Perennial grass	Accept	10
Poaceae	*Bambusa oldhamii*	Perennial grass	Accept	7
Poaceae	*Fargesia nitida*	Perennial grass	Accept	3
Poaceae	*Muhlenbergia capillaris*	Perennial grass	Accept	6
Primulaceae	*Cyclamen hederifolium*	Perennial dicot	Accept	5
Ranunculaceae	*Helleborus argutifolius*	Perennial dicot	Accept	6
Ranunculaceae	*Helleborus foetidus*	Perennial dicot	Accept	6
Rutaceae	*Geijera parviflora*	Tree/shrub	Accept	3
Sapindaceae	*Koelreuteria paniculata*	Tree	Needs Further Evaluation	13
Saxifragaceae	*Bergenia crassifolia*	Perennial dicot	Accept	5
Scrophulariaceae	*Leucophyllum frutescens*	Shrub	Accept	8
^**6**^ **TP**			**36**	
^**7**^ **FN**			**1**	
^**8**^ **NT**			**37**	
^**9**^ **Specificity for Non-IP**			**97%**	
^**10**^ **Accuracy for Non-IP**			**97%**	
^**11**^ **Overall Accuracy**			**95%**	

^1^TN = True negative

^2^FP = False positive

^3^IT = Total number of invasive species

^4^Sensitivity (accuracy for invasive species) = TN/(TN+FP)

^5^Accuracy for invasive species = (TN/IT) x 100

^6^TP = True positive

^7^FN = False negative

^8^NT = Total number of non-invasive species

^9^Specificity (Accuracy for non-invasive species) = TP/(TP+FN)

^10^Accuracy for Non-IP = (TP/NT) x 100

^11^Overall Accuracy (A_0_) = (TP+TN)/(IT+NT)

Typically, the testing of a risk assessment tool for accuracy includes post-hoc testing of a list of non-native species that are already known to be invasive versus non-native species that have been introduced for a substantial period of time into the area of concern, but are not considered invasive. We selected non-invasive species that had been in cultivation in California for at least 30 years with no record of escape and invasiveness. An accuracy rate can be determined by the number of correct predictions for the known invasive and non-invasive species. Overall accuracy is determined based on the equation: *A*_0_ = (*TP* + *TN*) / (*IT* + *NT*); where TP represents the true positives or the number of invasive species correctly rejected as high risk for invasiveness and TN represents the true negatives or the number of non-invasive species correctly accepted as low risk for invasiveness; IT is the total number of invasive species tested and NT is the total number of non-invasive species tested [38]. Invasive species accuracy or sensitivity is determined by using the equation *A*_*i*_ = *TP* / (*TP* + *FN*) where FN is the total number of false negatives or invasive plants that were correctly accepted as having low risk of invasiveness [31, 33, 38]. Finally, non-invasive species accuracy or specificity is *A*_*n*_ = *TN* / (*TN* + *FP*) where FP is the total number of false positives or non-invasive plants that were rejected for high risk for invasiveness. We analyzed the data in two ways, including 1) omitting plant species that were categorized as “needs further evaluation” (considering them to be neither a FP for invasive species nor a FN for non-invasive species), and 2) including “needs further evaluation” species as FP for invasive species or FN for non-invasive species.

The final score for each species was the sum for all of the scores for the all of the questions answered. Based on the separation in scores between the known invasive and non-invasive species, the scoring scale for the 19 question PRE tool was established to be <11 as an accept (low invasive risk), 11 to 13 was classified as “needs further evaluation”, and >13 as a reject (not allowed introduction due to its high invasive risk). Plants which fell into the “needs further evaluation” category may need additional assessment by an expert panel.

Likelihood ratio (LR) was used to compare the predictive potential of the questions we evaluated [31]. The LR assesses the proportion of rejected invaders to the proportion of rejected non-invaders using the equation LR = (TP/IT) / (FP/NT). LR is independent of the base-rate of invasives and non-invasives in the environment. Higher values give better predictive ability and a value of 1 would provide no predictive capability.

Receiver operating characteristic (ROC) curve analysis is commonly used to evaluate the performance of WRA tools [15, 33]. A ROC curve represents test sensitivity (accuracy for correctly categorizing non-invasive plants as having low risk of invasiveness) against the complement of specificity (accuracy for correctly categorizing invasive plants as having high risk of invasiveness) over the range of potential cut-off levels [39]. The area under the ROC curve (AUROC) is used to quantify the performance of a WRA test in discriminating between two classes (high risk versus low risk of invasiveness) [15]. The closer the AUROC is to 1, the higher the accuracy of the screening test, while an AUROC of 0.5 indicates the tool has low accuracy for correctly categorizing plant species as either high or low risk of invasiveness [40]. AUROC is calculated as the sum of trapeziums, or the difference between the x-values multiplied by half the sum of the y-values.

## Results and Discussion

### Development of the Plant Risk Evaluation (PRE) tool

In our initial evaluation of the 56 questions from existing WRA models, the cumulative scores (for all 56 questions) ranged from 21 to 44 for known invasive plants, with an average score of 31 (Table 1). The scores for known non-invasive plants ranged from 5 to 14, with an average score of 10. For each plant species screened, the percentage of questions answered for known invasive plants ranged from to 80% to 98%, with an average of 90%. The percentage of questions answered for known non-invasive plants ranged from to 86% to 95%, with an average of 89%.

The Fischer’s Exact Test identified a total of 31 questions that had a *P*>0.05 (S1 Table). The percentage of times each of the 56 questions was answered for known invasive plants ranged from 5% to 100%. The percentage of times each of the 56 questions was answered for known non-invasive plants ranged from to 0% to 100%. Eleven of the 56 questions were answerable less than 20% of the time both known invasive and non-invasive plants, so did not meet the question selection criteria. Of the 56 questions evaluated, 17 were eliminated because they did not provide statistically significant predictive power to separate known invasive from known non-invasive plants (S1 Table). Three questions were eliminated because of the inherent bias in the question. For example, the question was only known and answered when the phenomenon was studied, which was nearly always with known invasive species (i.e., allelopathy, palatability to animals, impacts on agricultural or grazing systems). Eight other questions showed statistical significance but were irrelevant to evaluating ornamental plants or new plant introductions. For example, “Has this species been exposed to a high level of domestication?” would not apply to new introductions and is primarily a question related to agricultural species. Species such as corn, which is highly domesticated, is known to have very low invasive potential [41]. Other irrelevant questions, such as “Does it easily establish within horticultural situations?” and “Are propagules dispersed by horticultural practices?” will always be answered yes for horticultural and ornamental species, as such species are expected to establish easily and are dispersed by humans through horticultural practices. Furthermore, the purpose of this model is not to evaluate the potential for significant impact or to prioritize species or populations for management or eradication, but rather to evaluate the potential to escape and establish outside of cultivation. Questions including, “Is this species found only in disturbed areas or is it also found in mature native vegetation as well?” and “Does this species negatively impact water relations on the site?” are included in other models designed to evaluation potential impacts [42, 43], but are not appropriate for the purpose of this model. Finally, there were three questions on the management of species that were eliminated from our final model. These included “Does it reshoot after all control methods?”, “Are effective herbicide treatment available?”, and “Does it need re-occurring control work several times per year?” Such questions do not provide information on the invasive potential of newly introduced ornamental plants, nor is there generally adequate information to answer these questions for ornamentals. These questions are more relevant to prioritization models that allow land managers a decision-making process for allocation of limited resources to manage invasive plant infestations [44].

Of the 19 questions we included in the final PRE tool, 11 showed statistical significance in separating known invasive from non-invasive species, four were the result of merging two similar questions, where both were significant or near significant (e.g., different methods of vegetative reproduction, various biotic and abiotic propagule dispersal mechanisms), one was the result of merging four similar questions (human-mediated propagule dispersal) that were significant or nearly significant (S1 Table). The remaining three questions included in the final PRE model included a new question on whether the plant produces viable seed. While there were other questions on the length of seed viability or whether seeds require pretreatment in order to germinate, these questions were often difficult to answer with ornamental species. Thus, we felt that a simpler form of the questions would be more useful and add additional power to the prediction of invasiveness and non-invasiveness. We retained one of the climate-related questions (Is the species found predominantly in a climate matching those within the targeted region?) because it is important for evaluating new plant introductions into a new geographic area. This question provided no separation in our analysis because we sampled from a population of known invasive and non-invasive species from within the geographic area of evaluation (i.e., California). We retained one question that had a *P>0*.*05*, which asks about the capability for fragments of the plant’s stems or roots to establish as new plants. We decided to retain this question to see whether it met our question selection criteria after further testing for the following reasons: 1) it represents an important aspect of vegetative reproduction for invasive plants; 2) we were already collecting information regarding vegetative reproduction for other questions; 3) it was answerable 100% of the time in our first round of testing; and 4) it had a relatively low *P*-value (*P = 0*.*2816*). The results of the additional screenings are presented in the next section.

### Accuracy testing of the Plant Risk Evaluation (PRE) Tool

In our evaluation of the 19 WRA questions, we were able to answer each question at a high frequency (similar to the same questions in the 56 question evaluation), ranging from a low of 54% for non-invasive plants regarding the question on the length of the juvenile period (question 15) to 100% for most other questions (Table 2). We were able to answer an average of 97% of the questions for both invasive and non-invasive plants (Table 3). For individual species, this ranged from 85 to 100% of the questions answered.

The results of the two-tailed Fischer’s Exact Test showed that 16 of the 19 questions were statistically significant in differentiating between the known invasive and non-invasive species. Furthermore, among those 16, the new question on viable seed (question 12) was highly significant (*P*<0.0001). In addition, the question on plant fragments capable of producing new plants (question 11), which was not significant at *P*<0.05 in the 56 question analysis, proved to be highly significant when evaluated with the final PRE tool on the 94 species (*P* = 0.0002). Of the remaining three questions, two were significant in the 56 question analysis, but were not significant at the *P*<0.05 level in the 19 question analysis. These included questions on whether seeds were quick to germinate (<2 months after dispersal) (question 14, *P* = 0.1296) and the length of the flowering period (question 16, *P* = 0.2320). Despite not being statistically significant at the *P*≤0.05 level, these questions did appear to contribute some level of separation between known invasive and non-invasive species. Finally, as was the case with the 56 question evaluation, the question asking if the species was found predominantly in a climate that matches those of the region of introduction (question 5) could not be evaluated with the 94 species because all the species assessed were already within the geographic area of evaluation (i.e., California).

While the scores for 16 of the 19 questions gave one point for a yes answer and zero points for a no answer, three questions were weighted more heavily (Table 2). In the climate matching questions (question 5) we used two points for a yes answer, which is consistent for other WRA models with the same question [15, 17]. However, for questions 1 to 3, we increased the weighting from one point for naturalization where it is not native (question 1), to two points for invasive elsewhere in the US or world (question 2), to three points for invasive elsewhere in the US or world, but in a similar climate (question 3). We considered that the risk of invasiveness in the region of interest was greater when a species had already become invasive in a different region of the world that had a similar climate to the region of interest.

**Fig 1:**
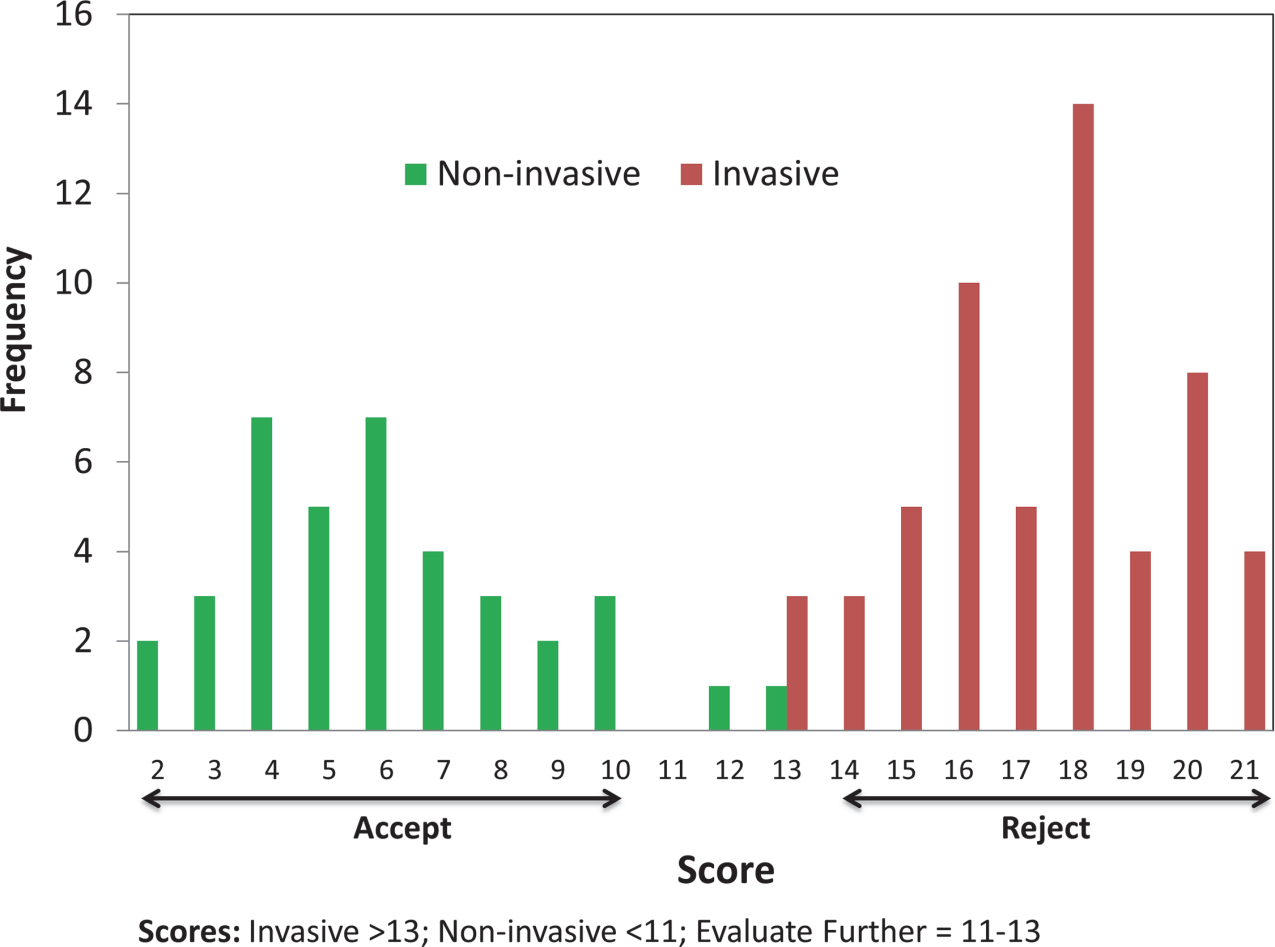
Histogram of scoring frequencies for the 19-question PlantRight plant risk evaluation (PRE) tool.

The scores for known invasive plants ranged from 12 to 21, while the scores for known non-invasive plants ranged from 2 to 13 (Table 3). By plotting score frequencies we were able to assign scoring cutoffs for the PRE tool (Fig. 1). From this plot we determined that scores below 11 were accepted as having a low risk of invasiveness, scores above 13 were rejected as having a high risk of invasiveness, and scores between 11 and 13 would be in the “needs further evaluation” category.

For the 57 known invasive plants evaluated through the 19 question PRE tool, no species were classified as accept (Table 3). Four species, however, were included in the “needs further evaluation” category, including *Carpobrotus chilensis*, *Elaeagnus angustifolia*, *Gleditsia triacanthos*, and *Retama monosperma*. All but *Gleditsia triacanthos* are listed by Cal-IPC as Moderately Invasive [6]. *Gleditsia triacanthos* is not listed by Cal-IPC as invasive, but is included in the *Weeds of California and Other Western States* as having escaped cultivation in California [45]. Thus, when species within the “needs further evaluation” category were excluded, the accuracy and sensitivity of the PRE tool in prediction invasiveness was 100%. Even when these four species were considered false positives (invasive species incorrectly accepted as non-invasive) the accuracy and sensitivity was 93%.

The 19 question PRE tool gave no false negatives (non-invasive species rejected as invasive), but did classify one species (*Koelreuteria paniculata*) in the “needs further evaluation” category (Table 3). *Koelreuteria paniculata* has been reported to have escaped cultivation and become invasive in several US states, particularly Texas [46] and Florida [47], though it has not been reported as an invasive or naturalized plant in California. Thus, the percent accuracy or specificity of the model when plants classified as “needs further evaluation” are excluded is 100%. Even when the “needs further evaluation” species are considered as false negatives, the accuracy is still a very high 97%. When considering both known invasive and non-invasive species, the overall accuracy of the PRE model was 100% when “needs further evaluation “species were excluded and 95% when they were included.

Smith et al. [31] noted that the likelihood ratio (LR) can be useful in assessing the proportion of rejected invader to the proportion of rejected non-invaders and is independent of the base-rate of invasives and non-invasives in the environment. In this case, values close to one provide no predictive power in categorizing potential invasive from non-invasive species. The LR value for the PRE tool demonstrated a very high value even when including “needs further evaluation” species (5.84). When not including “needs further evaluation” the LR value was infinity as the denominator was zero.

**Fig 2:**
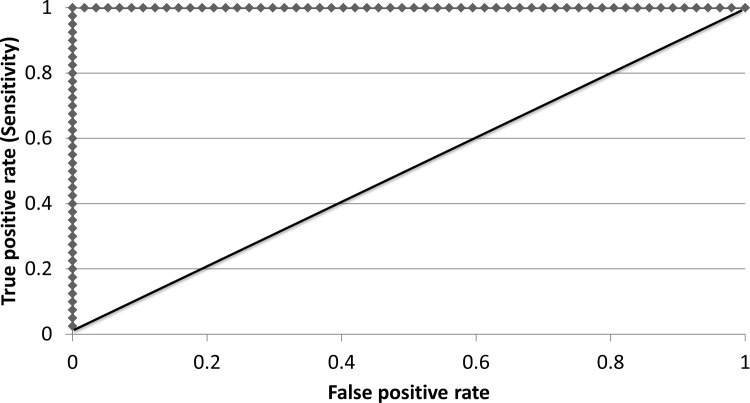
Receiver operating characteristic (ROC) curve analysis 19-question PlantRight plant risk evaluation (PRE) tool.

For the ROC curve analysis, a positive result was considered anything above the threshold for “needs further evaluation” (>10) and a negative result was an “accept” (<11) outcome. As further support for the accuracy of the PRE tool, the AUROC evaluation had a score of 1, indicating it has high accuracy for correctly categorizing plant species as either high or low risk of invasiveness (Fig. 2).

We believe that the increase in accuracy with the PRE model compared to other similar models may be due to both the reduction in subjectivity associated with questions that were changed or removed, as well as the elimination of questions that added no separation power. It is possible that the inclusion of these questions reduced the ability to accurately evaluate non-invasive plants.

## Conclusions

The next steps in the development and validation of the PlantRight PRE tool will be to: 1) test the consistency of the tool by different users, 2) test the accuracy of the tool in evaluating invasive risk on a national scale (to demonstrate that it can be used beyond California, and at different scales), 3) incorporate climate matching capabilities, 4) develop an online database in partnership with UC Davis, and 5) encourage nursery industry adoption. The PRE tool can be used preventatively by the nursery industry to screen ornamental plants for potential invasiveness prior to introduction to the marketplace. The PlantRight PRE tool can also give a prediction of the risk of invasiveness (low or high) for a given species at a designated location. The tool is expected to provide the industry with multiple potential benefits, including: 1) an online service utilizing the PlantRight PRE tool to accurately assess invasive risk early in the evaluation process (before making a significant economic investment), 2) additional information regarding taxonomy, reproductive characteristics, culinary and medicinal uses, patent information and more, 3) optional services including maps of CLIMEX climate-matching results under various assumptions (e.g., drought tolerance) and scenarios (e.g., irrigation, climate change), and 4) a tiered-access/password-protected PlantRight PRE database, with both a public database of PREs and password-protected PREs for industry clients (to protect their intellectual property).

Because invasive plants represent only a small percentage of the horticultural inventory (~1%), screening plants for invasive qualities should not present a major economic hardship to the industry. Pre-screening of potential introductions would be expected to categorize the vast majority of species as possessing low invasive potential, and identify relatively few as having a high probability of becoming invasive [1]. More importantly, because development of new cultivars represents a significant economic investment for nursery growers throughout the US, pre-screening would prevent nurseries from spending important research dollars to develop new cultivars with high invasive potential.

## Supporting Information

S1 Table Questions used to develop the PRE model, including the statistical comparisons between invasive and non-invasive species for each questions, the percent each questions was answered and whether the questions was eliminated or added to the final model. (PDF)
