# Supplementary material for: The Development of a Plant Risk Evaluation (PRE) Tool for Assessing the Invasive Potential of Ornamental Plants
Source: PLoS One. 2015 Mar 24;10(3):e0121053. doi: 10.1371/journal.pone.0121053 (PMC4372384; doi:10.1371/journal.pone.0121053)
Supplement: S1 Table — (PDF) [file pone.0121053.s001.pdf]

Table S1. Questions used to develop the PRE model, including the statistical comparisons between invasive and non-invasive species for each questions, the percent each questions was answered and whether the questions was eliminated or added to the final model. Entries in bold indicate questions included in final PRE model and reasons for inclusion. Brackets after question indicate citation where question is included in WRA model.

56 Questions tested for PRE tool	Fisher's Exact Test (2-tail)	% invasive plant Q was answered	% non-invasive plant Q was answered	Is question in the 19-Q PRE tool?	Question # in the 19-Q PRE tool	Reason for inclusion or omission in 19-Q final PRE model
**Has the species become naturalized where it is not native?[15],[17],[27],[33]**	*P*<0.0001*	100	100	Yes	1	**Statistically significant.**
Is the plant noted as being invasive in California?[2],[15],[16],[27],[33]	*P*<0.0001*	100	100	No	-	Model developed for entire country. Question no longer appropriate.
**Is the plant noted as being invasive elsewhere in the US?[2],[15],[16],[27],[33]**	*P*<0.0001*	100	100	Yes	3	**Statistically significant. Changed to include US or world in similar climatic region.**
**Is the plant noted as being invasive elsewhere in the world?[2],[15],[16],[33]**	*P*<0.0001*	100	100	Yes	2	**Statistically significant. Changes to include US or world.**
**Are other species of the same genus invasive in other areas with a similar climate?[2],[15],[16],[33]**	*P*=0.0006*	95	100	Yes	4	**Statistically significant.**
Has this species been exposed to a high level of domestication?[15],[17],[33]	*P*=0.0002*	100	100	No	-	Question not relevant to newly introduced horticultural species.
Does it easily establish within horticultural situations?[16],[27]	*P*=0.0689	100	100	No	-	Question not relevant as model is designed to evaluate horticultural species.

Is this species found only in disturbed areas or is it also found in mature native vegetation as well? (shade tolerant)[15],[17]	*P*=0.0007*	95	100	No	-	Question not relevant to newly introduced horticultural species.
Does it reshoot after all control methods? (cutting, pulling, herbicide)[16],[17]	*P*<0.0001*	100	100	No	-	Not relevant to plants that have yet to be introduced.
Are effective herbicide treatment available?[15],[17]	*P*<0.0001*	100	100	No	-	Not relevant to plants that have yet to be introduced.
Does it need re-occurring control work several times per year? (high seeding annual, persistent seeds, and/or rapid maturation of seedlings missed from previous treatments)[15],[16],[17]	*P*<0.0001*	100	100	No	-	Not relevant to plants that have yet to be introduced.
**Does it dominate in areas it has already invaded (displaces natives)?[15],[16],[27],[33]**	*P*<0.0001*	100	100	Yes	6	**Statistically significant. Merged with other similar question.**
**Can it overtop and/or smother surrounding vegetation?[15],[16],[17],[33]**	*P*=0.0006*	100	100	Yes	6	**Statistically significant. Merged with other similar question.**
Is the plant noted as being allelopathic and/or alter soil/water conditions?[15],[17],[33]	*P*=0.0006*	100	100	No	-	Biased answers as allelopathy is primarily studied in invasive plants.
**Is the plant a health risk to humans or animals/fish? (Toxic tendencies)[15],[16],[17],[27],[33]**	*P*=0.0002*	100	100	Yes	8	**Statistically significant.**
**Does the plant produce impenetrable thickets, blocking or slowing movement?[15],[16],[17],[33]**	*P*=0.0006*	100	100	Yes	9	**Statistically significant.**

**Is the plant noted as being highly flammable and/or promotes fire and/or changes fire regimes?[15],[16],[17],[33]**	*P*=0.0015*	100	100	Yes	7	**Statistically significant.**
Does this species negatively impact water relations on the site?[15],[17],[27],[33]	*P*=0.0118*	100	100	No	-	Not relevant to plants that have yet to be introduced.
Does the plant fix nitrogen?[15],[16],[27],[33]	*P*=0.5059	100	100	No	-	Not statistically significant.
Is the plant considered habitat or a food source for invasive invertebrates and animals?[15],[17],[33]	---	19	0	No	-	Not answered enough to conduct analysis.
Does this species hybridize with a native species?[15],[17],[33]	*P*=1.0000	67	100	No	-	Not statistically significant.
If the pollinators are known, could this species distract the necessary pollinators from native plants?[15],[17],[33]	---	5	0	No	-	Not answered enough to conduct analysis.
Is the plant known to be unpalatable to grazers?[15],[17],[33]	*P*<0.0001*	90	100	No	-	Biased as answer for horticultural species is based on lack of information. Not statistically significant.
Has the species been noted as impacting agricultural/grazing systems via reduced crop yield?[15],[17],[27]	*P*=0.0051*	100	100	No	-	Biased as answer for horticultural species as little is known about their impacts and answer is always no.
**Are seed quick to germinate?[2],[17]**	*P*=0.0017*	71	100	Yes	14	**Statistically significant.**
Does it reproduce vegetatively?[2],[17]	*P*=1.0000	100	100	No	-	Not statistically significant.

If so, it is via rhizomes?[2],[17]	*P*=0.6853	100	100	No	-	Not statistically significant.
If so, is it via stolons?[2],[17]	*P*=0.6350	100	100	No	-	Not statistically significant.
**If so, is it via root sprouts/suckers?[2],[17]**	*P*=0.7115	100	100	Yes	10	**Merged with similar question.**
If so, it is via layering?[2],[17]	*P*=1.0000	100	100	No	-	Not statistically significant.
**If so, it is via stem/trunk sprouts/coppicing?[2],[15]**	*P*=0.0556	100	100	Yes	10	**Provides some separation. Merged with other similar question.**
**Are plant fragments ~~are~~ capable of producing new plants?[2],[17]**	*P*=0.2816	100	100	Yes	11	**Showed statistical significance in subsequent evaluation of 19 question tool ~~with additional species~~.**
Do plants reproduce more than once a year?[2],[17]	*P*=0.6067	81	100	No	-	Not statistically significant.
**Is there a short juvenile period, such that seeds are produced in first three years (herbaceous) or in first five years (woody)?[2],[17]**	*P*=0.0111*	100	93	Yes	15	**Statistically significant.**
Are 'volunteer' seedlings commonly seen nearby mature plants in gardens or landscapes? ~~Are volunteer seedlings common?~~ [16],[17]	*P*=0.1667	81	86	No	-	Not statistically significant.
**Do plants produce copious viable seeds each year (>1000)?[2],[17]**	*P*=0.0091*	90	100	Yes	13	**Statistically significant.**
Is the plant self-compatible? (capable of self-pollination)[2],[17]	*P*=1.0000	57	43	No	-	Not statistically significant or answered with enough frequency.

Is the seed produced via cross-pollination as well as self?[2],[15],[17]	*P*=1.0000	24	7	No	-	Invasive and non-invasive species not answered enough to conduct analysis.
**Is flowering time long, such that seed production is more than 3 months each year?[2],[17]**	*P*=0.0011*	100	100	Yes	16	**Statistically significant.**
Do seeds remain viable in soil for more than three years?[15],[17]	*P*=0.1357	76	21	No	-	Not statistically significant and not answered with enough frequency in non-invasives.
Do the seeds require pretreatment in order to germinate?[2],[17]	*P*=1.0000	67	7	No	-	Invasive species not answered enough to conduct analysis.
**Are propagules dispersed by mammals/insects or birds?[15],[16],[17]**	*P*=0.0016*	100	100	Yes	17	**Statistically significant. Merged with other animal dispersal question.**
**Are propagules dispersed by wind?[15],[16],[17]**	*P*=0.0313*	100	100	Yes	18	**Statistically significant. Merged with water question.**
**Are propagules dispersed by water?[15],[16],[17]**	*P*=0.0003*	100	100	Yes	18	**Statistically significant. Merged with wind question.**
Are propagules dispersed by exploding seed pods?[15],[17]	*P*=0.5059	100	100	No	-	Not statistically significant.
**Are propagules dispersed by domestic animals?[15],[16],[17]**	*P*=0.0022*	100	100	Yes	17	**Statistically significant. Merged with animal dispersal question.**
**Are propagules dispersed by contaminated seed?[15],[16],[17],[33]**	*P*=0.0689	100	100	Yes	19	**Provides some separation. Merged with other dispersal questions.**
**Are propagules dispersed by farm equipment, vehicles or boats?[15],[16],[17],[33]**	*P*=0.0006*	100	100	Yes	19	**Statistically significant. Merged with other dispersal questions.**

Are propagules dispersed by horticultural practices?[15],[16],[17],[33]	*P*=0.0280*	100	100	No	-	Not relevant as model was designed to evaluate horticultural plants.
**Are propagules dispersed by agriculture practices?[15],[16],[17],[33]**	*P*=0.0118*	100	100	Yes	19	**Statistically significant. Merged with other dispersal questions.**
**Are propagules dispersed by clothing/shoes?[15],[16],[17],[33]**	*P*=0.0118*	100	100	Yes	19	**Statistically significant. Merged with other dispersal questions.**
Does the species possess underground storage organs?[16],[17]	*P*=0.2087	95	50	No	-	Not statistically significant.
Does the species require a pollinator that is not available or plentiful in the area of introduction?[16],[17]	--	10	7	No	-	Not answered with enough frequency.
Is the plant known to demonstrate a rapid growth rate?[16],[17]	*P*=0.0867	95	64	No	-	Not statistically significant.
**Is the species found predominately in a climate that matches those within the targeted region?[15],[17],[27]**	--	100	100	Yes	5	**More important question when evaluating new introductions.**
Is the species able to thrive in many climate units? Does it have a large native latitudinal range? (5 USDA hardiness zones)[15],[17],[27]	---	100	100	No	-	No variation in answers between invasive and non-invasive species.

[a] Entries in bold indicate questions included in final PRE model and reasons for inclusion.

[b]Brackets after question indicate citation where question is included in WRA model.
